# Kdm7aa Orchestrates an Immunomodulatory Cardiomyocyte Program to Enable Zebrafish Heart Regeneration

**DOI:** 10.3390/ijms262010044

**Published:** 2025-10-15

**Authors:** Weibin Lin, Yuan Shi, Jin Tian, Xinru Liu, Fubin Weng, Zekai Wu

**Affiliations:** 1 Key Laboratory of Gastrointestinal Cancer (Fujian Medical University), Ministry of Education, Fuzhou 350122, China; 2 State Key Laboratory of Mariculture Breeding, College of Marine Sciences, Fujian Agriculture and Forestry University, Fuzhou 350002, China

**Keywords:** heart regeneration, zebrafish, *kdm7aa*, immune response, chemokines

## Abstract

Myocardial infarction triggers limited repair in adult mammals but robust regeneration in zebrafish. Epigenetic regulation and immune responses are recognized as critical for successful regeneration. However, the molecular links between these processes have not been fully elucidated. By performing single-cell RNA sequencing of zebrafish ventricular cardiomyocytes after injury, we identified a regeneration-induced immunomodulatory cluster that specifically expressed the histone demethylase gene *kdm7aa*. Functional perturbations, including CRISPR/Cas9-mediated *kdm7aa* mutation and pharmacological inhibition of Kdm7aa activity using TC-E5002, impaired cardiac regeneration. Bulk RNA sequencing showed that *kdm7aa* drives an inflammatory transcriptional program, prominently activating chemokines such as *cxcl8a* and *cxcl19* that coordinate immune cell recruitment. Cross-species analyses revealed injury-induced Kdm7a upregulation in regeneration-competent neonatal mouse hearts but not in adult mouse or human hearts. These data identified Kdm7aa as a regeneration-induced epigenetic regulator that enabled cardiomyocytes to adopt a transient immune-activating phenotype, linking histone demethylation to chemokine signaling and suggesting a potential therapeutic strategy to enhance mammalian cardiac repair.

## 1. Introduction

Myocardial infarction (MI), primarily caused by acute myocardial ischemia resulting from coronary artery occlusion, is the leading cause of mortality worldwide. Although reperfusion and other interventional therapies mitigate early morbidity, heart transplantation is still the only efficient treatment for end-stage MI [1]. Therefore, identifying strategies to promote intrinsic cardiac repair following injury holds significant potential for substantially reducing MI-related mortality. Unlike adult mammals, zebrafish (*Danio rerio*) retains a striking capacity to regenerate myocardium throughout its life [2]. Elucidating the regulatory mechanisms underlying zebrafish heart regeneration offers valuable insights and novel avenues to stimulate cardiac regeneration in mammals.

Epigenetic modifications, which regulate gene expression without altering the DNA sequence, have recently become a major focus of research. Among these modifications, the methylation of histone lysine residues is particularly pervasive and governs key biological processes, including transcriptional regulation, translation, and the maintenance of genomic stability [3]. The methylation state of histone lysine is dynamically regulated by lysine methyltransferases (KMTs) and lysine demethylases (KDMs), and whether the modification leads to gene activation or repression depends on both the specific lysine residue that is modified and the degree of methylation [4]. In general, methylations at histone H3 lysine 4 (H3K4), H3K36, and H3K79 are associated with transcriptional activation, whereas methylations at H3K9 and H3K27 are linked to transcriptional repression [3]. Extensive evidence implicates mutation or dysregulation of KMTs, KDMs, and methyl-lysine reader proteins results in a wide spectrum of diseases, underscoring their value as therapeutic targets [5]. Accordingly, precise modulation of histone lysine methylation during cardiac regeneration may constitute a pivotal strategy for enhancing myocardial repair.

Beyond the modulation of gene expression by epigenetic regulators such as histone demethylases, a growing body of evidence highlights the indispensable role of the immune response in facilitating successful cardiac regeneration in zebrafish [6]. The recruitment of specific immune cells, particularly macrophages and neutrophils, to the injury site in a timely manner is essential in this process [7]. Studies demonstrated that chemokines, such as Cxcl8 and Ccl12, were crucial for orchestrating this immune cell influx following cardiac damage [7]. Furthermore, experimental depletion of macrophages, or genetic disruption of key recruitment signals, consistently led to impaired cardiomyocyte proliferation, defective debris clearance, excessive fibrotic scarring, and, ultimately, a failure of functional regeneration [8]. This compelling evidence strongly supported the paradigm that immune cell infiltration is not merely a consequence of injury but an essential prerequisite for the complex cellular and molecular events driving myocardial repair in zebrafish. Understanding the precise mechanisms governing immune cell recruitment and function, therefore, represents a critical facet in deciphering the innate regenerative capacity of the zebrafish heart.

Building on our earlier single-cell RNA-sequencing analysis of major cardiac cell types during zebrafish heart regeneration [9], we re-examined the expression dynamics of ventricular cardiomyocytes. This re-analysis uncovered a regeneration-specific subpopulation of ventricular cardiomyocytes, which showed robust expression of the histone demethylase *kdm7aa*. Functional studies demonstrated that *kdm7aa* is indispensable for cardiac regeneration, as it initiated an innate immune response by transcriptionally activating chemokine genes, including *cxcl8a* and *cxcl19*. Additionally, the regeneration-associated up-regulation of *Kdm7a* was also observed in neonatal mice, which retain regenerative competence. However, this increase was absent in adult mice and humans, both of which lacked regenerative capacity. Taken together, our findings established a mechanistic link between *kdm7aa* and chemokine-driven immune activation during zebrafish heart regeneration and hypothesized that Kdm7aa enables a transient immune-activating state in cardiomyocytes required for regeneration, thereby offering novel molecular targets and conceptual insights for enhancing cardiac regeneration in mammals.

## 2. Results

### 2.1. Single-Cell Transcriptomics Reveals Distinct Ventricular Cardiomyocyte Populations and Regeneration-Specific Subsets

**Figure 1:**
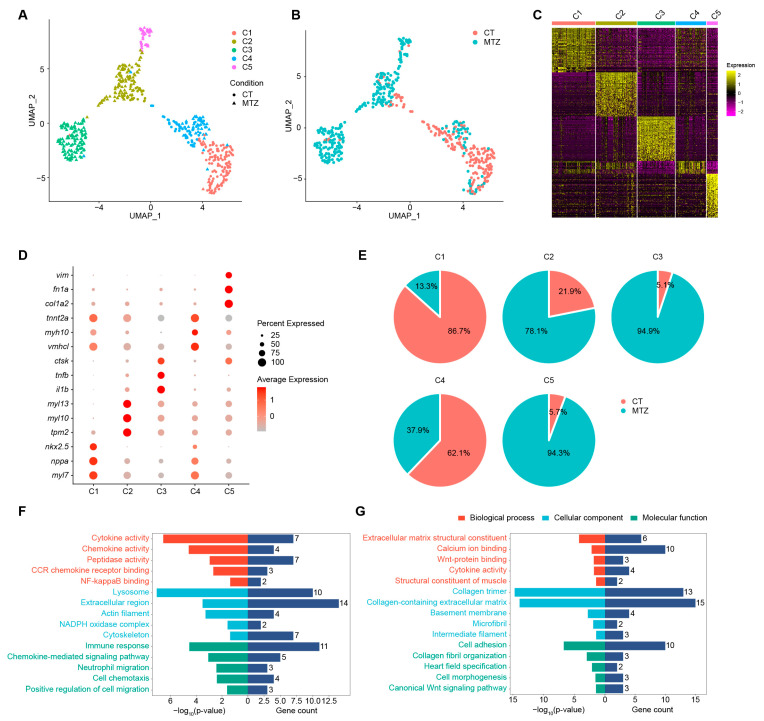
Single cell RNA sequencing revealed molecular diversity of ventricular cardiomyocytes during zebrafish heart regeneration. (**A**,**B**) UMAP analysis of ventricular cardiomyocytes from both untreated (CT) and MTZ-treated zebrafish embryos, colored by clusters and shaped by sample conditions (**A**), or colored by sample conditions (**B**). (**C**,**D**) Heatmap (**C**) and bubble diagram (**D**) showing the expression of marker genes in each cluster from (**A**). (**E**) Pie charts showing the constituent proportions of treatment conditions in different clusters. (**F**,**G**) Representative GO terms of specifically expressed genes in C3 (**F**) and C5 (**G**).

To achieve selective ablation of ventricular cardiomyocytes (CM-V), we employed *Tg(vmhc:mCherry-NTR; amhc:EGFP)* double-transgenic zebrafish, in which MTZ treatment induces nitroreductase-mediated cell death only in mCherry-labeled CM-V. Dual fluorescence reporters enabled simultaneous tracking of ventricular (mCherry) and atrial (EGFP) cardiomyocytes during subsequent heart regeneration. In an earlier study we performed single-cell RNA sequencing (scRNA-seq) on the five major cardiac cell types: atrial cardiomyocytes (CM-A), CM-V, endothelial cells (EC), epicardial cells (EP) and epicardial-derived cells (EPDC) after ventricular ablation, alongside untreated controls [9]. In order to explore the ventricular-specific molecular programs underlying regeneration, we extracted all CM-V profiles from the previously generated scRNA-seq dataset for further analysis. UMAP of the CM-V transcriptomes resolved five distinct sub-clusters (Figure 1A,B). Marker-gene analysis assigned unique molecular identities to each cluster (Figure 1C,D). Cluster 1 (C1) exhibited a transcriptional profile of immature cardiomyocytes, characterized by robust expression of early cardiac markers including *nkx2.5*. Clusters 2 (C2) and 4 (C4) were enriched for sarcomeric genes such as *myl10* and *tnnt2a*, likely representing mature contractile cardiomyocytes. Cluster 3 (C3) was characterized by the specific expression of pro-inflammatory cytokines and chemokines, including *il1b* and *tnfb*, suggesting an immunomodulatory phenotype. Cluster 5 (C5) uniquely expressed extracellular-matrix and fibrosis-related genes, including *fn1a* and *vim*, indicative of a remodeling state. Quantitative assessment of cluster composition revealed that clusters C3 and C5 were significantly enriched in injured hearts compared to uninjured controls, indicating them as regeneration-specific cell populations (Figure 1E). Moreover, these results suggested that the inflammatory program in C3 and the remodeling program in C5 were essential for zebrafish ventricular repair.

GO enrichment analysis of marker genes from the regeneration-specific clusters C3 and C5 delineated two distinct functional programs. C3 exhibited significant enrichment of innate-immune and inflammatory processes, such as cytokine and chemokine activity, NF-κB binding, immune response and cell chemotaxis, which coordinated leukocyte recruitment after injury (Figure 1F). In contrast, C5 was enriched for extracellular-matrix (ECM) organization and developmental terms, including ECM structural constituent, collagen trimers, collagen-containing ECM, cell adhesion, and heart-field specification, indicating a fibrogenic role accompanied by the reactivation of embryonic pathways (Figure 1G). Collectively, these data identified two regeneration-specific cardiomyocyte populations: C3 orchestrated the inflammatory milieu necessary for regeneration, whereas C5 promoted ECM remodeling and development-related gene programs to stabilize and rebuild the injured zebrafish ventricle.

### 2.2. KDM7aa Is a Regeneration-Induced Epigenetic Regulator Expressed in the Immunomodulatory Cluster

Growing evidence indicates that epigenetic reprogramming is indispensable for cardiac regeneration because it enables the rapid transcriptional shifts that drive cardiomyocyte proliferation, inflammation, and tissue remodeling [10]. Among epigenetic regulators, KDMs remove histone marks and are thus well positioned to mediate this regenerative plasticity [4]. However, the individual KDM family members involved and the specific cellular contexts where they function during zebrafish heart regeneration remain poorly understood.

**Figure 2:**
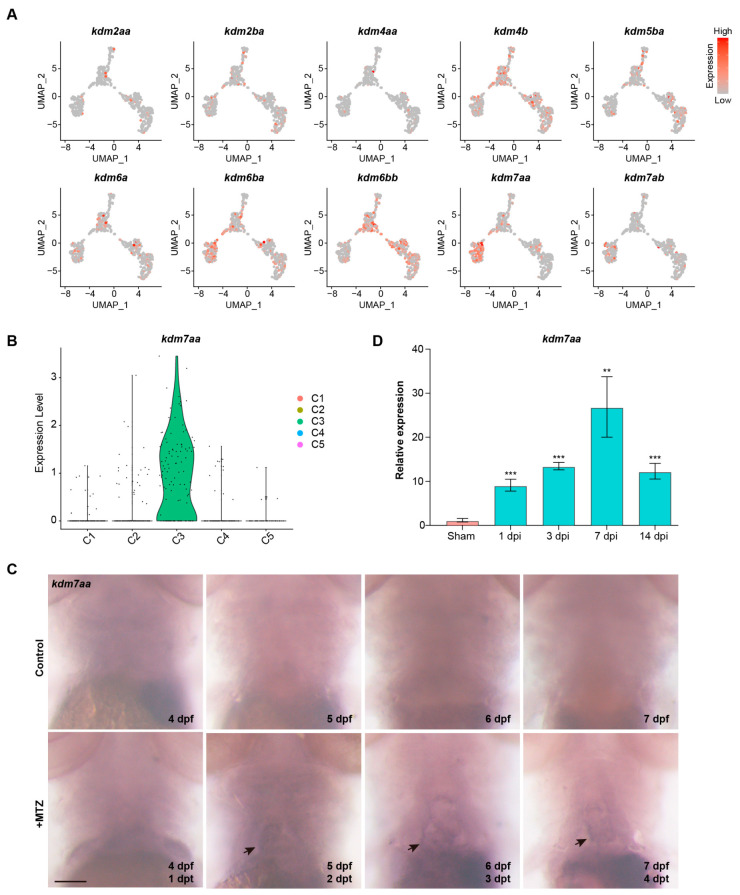
*kdm7aa* was induced during zebrafish heart regeneration. (**A**) UMAP projections showing expression patterns of KDM family genes. (**B**) Violin plot depicting *kdm7aa* expression levels across ventricular cardiomyocyte subclusters. (**C**) Whole-mount in situ hybridization for *kdm7aa* in *Tg(vmhc:mCherry-NTR; amhc:EGFP)* transgenetic zebrafish hearts during normal development and heart regeneration. Black arrow indicates expression of *kdm7aa* in cardiac region. Scale bar, 100 μm. (**D**) qRT-PCR analysis of *kdm7aa* expression during adult zebrafish heart regeneration. N = 3. One-way ANOVA analysis, ** *p* < 0.01, *** *p* < 0.001.

To identify the exact lysine demethylases involved in cardiac repair, we examined the expression of all KDM family genes in ventricular cardiomyocytes using our single-cell transcriptomic dataset. The results showed that *kdm7aa* was highly expressed in C3, the immune-responsive cardiomyocyte subset enriched during regeneration, suggesting that *kdm7aa* may regulate the inflammatory programs in this population (Figure 2A,B). Whole-mount in situ hybridization confirmed the spatial restriction of *kdm7aa* (Figure 2C). *kdm7aa* transcripts were undetectable in the uninjured heart but became apparent within the cardiac region after MTZ treatment. To verify whether the *kdm7aa* is also involved in cardiac regeneration of adult zebrafish, we examined its expression at various time points after ventricular cryoinjury. *kdm7aa* mRNA rose significantly at 1 dpi, continued to increase to maximum at 7 dpi, and then declined at 14 dpi (Figure 2D). Together, these findings identify *kdm7aa* as a regeneration-induced epigenetic regulator, implying its role in regulating immune pathways required for effective repair.

**Figure 3:**
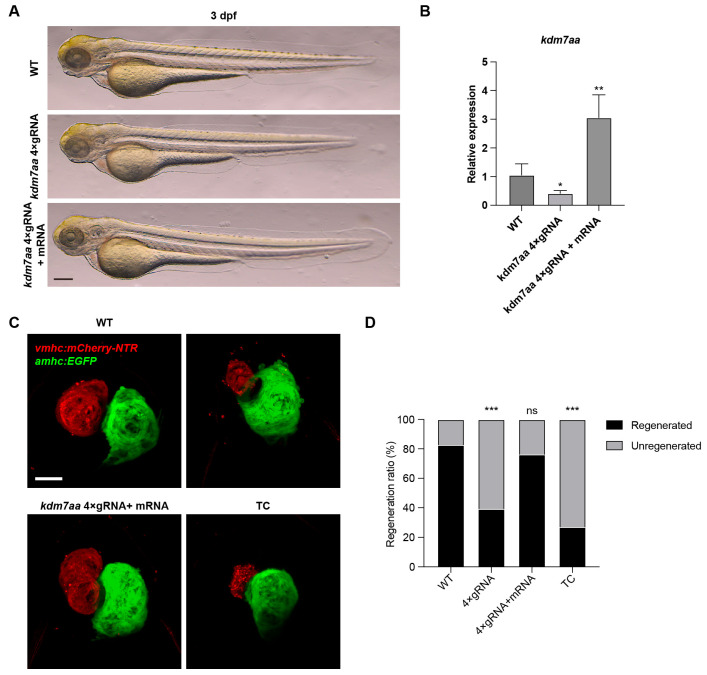
Kdm7aa is a key regulator of zebrafish heart regeneration. (**A**) Bright filed image of *kdm7aa* knockdown and overexpressed embryos. Scale bar, 200 μm. (**B**) qRT-PCR analysis of *kdm7aa* in knockdown and overexpressed embryos. N = 4. Student’s *t* test. *, *p* < 0.05, **, *p* < 0.01. (**C**) Maximum intensity projections at 4 dpt in the *Tg(vmhc:mCherry-NTR; amhc:EGFP)* transgenic background for four groups: wildtype, *kdm7aa* mutant, *kdm7aa* mutant injected with *kdm7aa* mRNA, and TC-E5002 treated wildtype. Scale bar, 100 μm. (**D**) Percentage of hearts exhibiting successful regeneration in each group in (**A**). N = 98, 92, 88, 93, respectively. Chi-square analysis was used for statistical analysis. ns, not significant, ***, *p* < 0.001.

To functionally validate the role of *kdm7aa* in regeneration, we developed *kdm7aa* F0 chimeric mutants using the CRISPR-Cas9 system by co-injecting four target-specific sgRNAs into single-cell zebrafish embryos, as previous reported [11]. qRT-PCR analysis showed that the injection of sgRNAs could significantly downregulate the expression level of *kdm7aa*, without developmental defects (Figure 3A,B). Ventricular injury was induced by treating 3 dpf zebrafish embryos with MTZ. The *kdm7aa* mutants exhibited a significant impairment in cardiac regeneration (Figure 3C,D). The co-injection of wild-type *kdm7aa* mRNA could increase its expression level, and partially ameliorated this regenerative defect (Figure 3C,D). Furthermore, treatment with the KDM7 inhibitor TC-E5002 phenocopied the *kdm7aa* mutants, further underscoring *kdm7aa* as an essential epigenetic regulator for heart repair in zebrafish (Figure 3C,D).

### 2.3. Transcriptomic Profiling Reveals kdm7aa-Dependent Regulation of Immune Pathways During Regeneration

**Figure 4:**
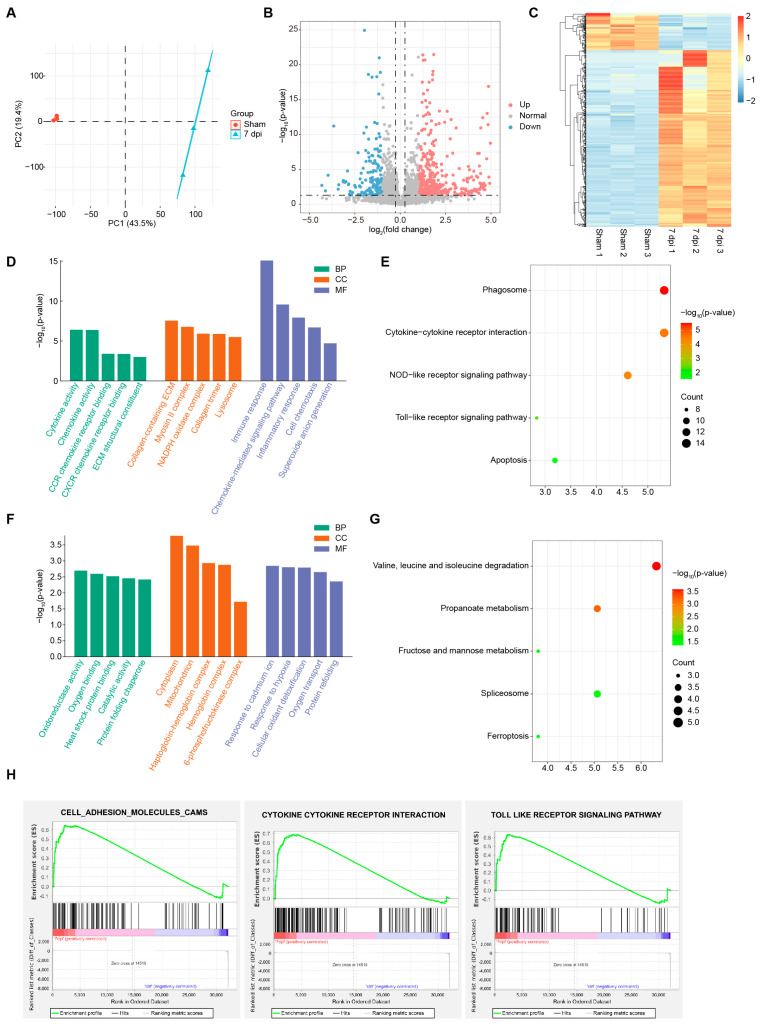
Global transcriptomic changes during zebrafish heart regeneration. (**A**) PCA analysis of RNA-seq data showing transcriptional separation between hearts at 7 dpi and sham-operated controls. (**B**,**C**) Volcano plot (**B**) and heatmap (**C**) showing differentially expressed genes between 7 dpi and sham-operated hearts. (**D**,**E**) Representative GO (**D**) and KEGG (**E**) terms enriched among genes upregulated during heart regeneration. (**F**,**G**) Representative GO (**F**) and KEGG (**G**) terms enriched among genes downregulated during heart regeneration. (**H**) GSEA analysis showing signaling pathways upregulated during heart regeneration.

To characterize the molecular regulatory network governed by *kdm7aa* during cardiac regeneration, we performed bulk RNA-seq on zebrafish ventricles collected from sham-operated controls, cryoinjured hearts at 7 dpi, and cryoinjured hearts exposed to the KDM7 inhibitor TC-E5002 at 7 dpi. PCA revealed clear transcriptomic divergence between sham and 7 dpi samples, underscoring the extensive transcriptional reprogramming that accompanies regeneration (Figure 4A). Differential-expression analysis identified 592 genes whose expression changed by more than two-fold (*p* value < 0.05), comprising 489 up-regulated and 103 down-regulated genes (Figure 4B,C). Up-regulated genes were significantly enriched for immune and ECM modules (Figure 4D,E). GO terms included cytokine activity, chemokine activity, collagen-containing ECM and immune response, whereas KEGG pathways highlighted cytokine–cytokine receptor interaction and Toll-/NOD-like receptor signaling. These signatures recapitulated the functional categories that defined the regeneration-specific clusters identified in our single-cell RNA-seq dataset. In contrast, the down-regulated gene set was dominated by metabolic pathways, including oxidoreductase activity, catalytic activity, mitochondrion, propanoate metabolism, and fructose and mannose metabolism (Figure 4F,G). GSEA also showed the activation of immune response and cytokine signaling modules at 7 dpi, thereby reinforcing the central role of *kdm7aa* in coordinating the inflammatory milieu required for effective cardiac repair (Figure 4H).

**Figure 5:**
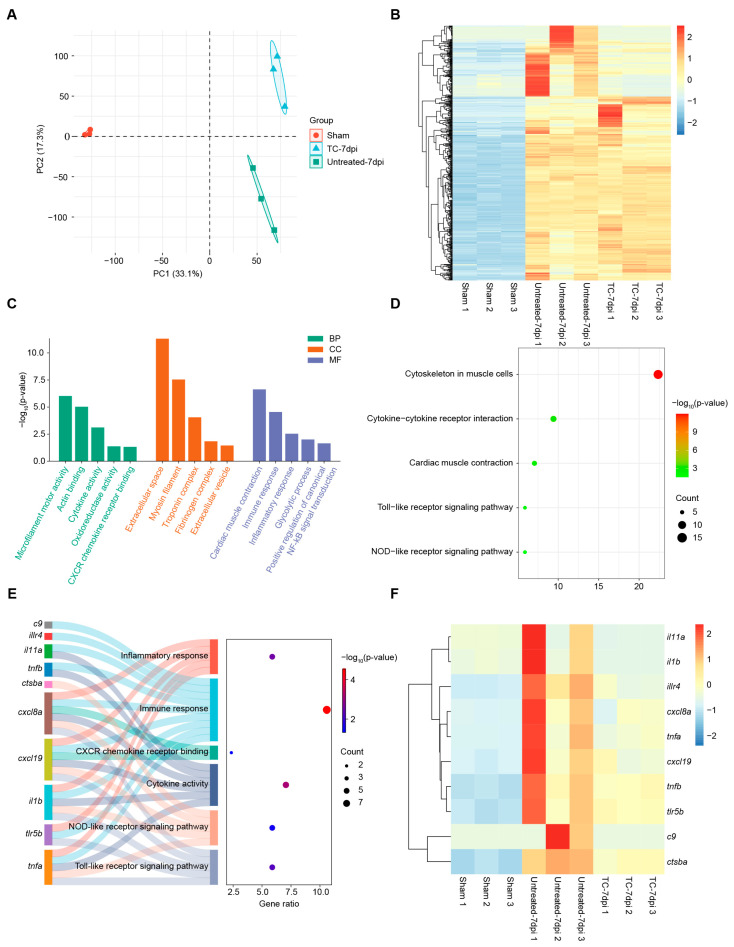
Transcriptomic analysis of Kdm7aa inhibition during zebrafish heart regeneration. (**A**) PCA analysis of RNA-seq data showing the heterogeneity among sham-operated hearts, hearts at 7 dpi and hearts at 7 dpi treated with TC-E5002. (**B**) Heatmap displaying the gene set upregulated at 7 dpi, with expression shown across sham-operated hearts, 7 dpi hearts, and 7 dpi hearts treated with TC-E5002. (**C**,**D**) Representative GO (**C**) and KEGG (**D**) terms enriched among genes that were upregulated during heart regeneration and suppressed after TC-E5002 treatment. (**E**,**F**) Sankey diagram (**E**) and heatmap (**F**) highlighting genes associated with immunomodulatory GO and KEGG terms shown in (**C**,**D**).

**Figure 6:**
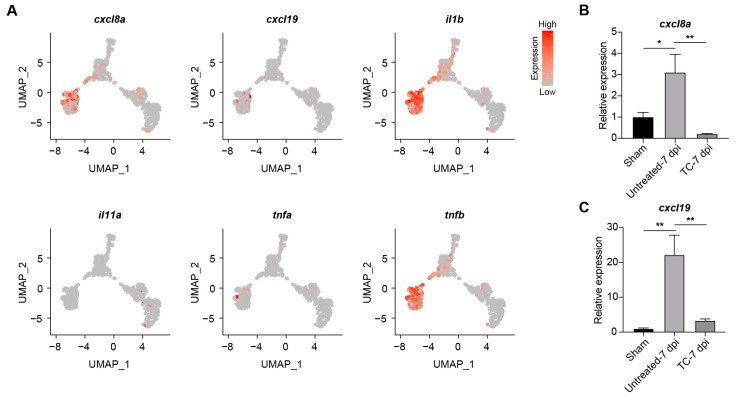
*cxcl8a* and *cxcl19* function downstream of *kdm7aa* during zebrafish heart regeneration. (**A**) UMAP projections showing expression patterns of immunomodulatory ligands associated with kdm7aa inhibition. (**B**,**C**) qRT-PCR analysis of *cxcl8a* and *cxcl19* expression in sham-operated hearts, hearts at 7 dpi and hearts at 7 dpi treated with TC-E5002. N = 3. One-way ANOVA analysis, * *p* < 0.05, ** *p* < 0.01.

Integrative transcriptomic profiling of sham-operated ventricles, cryoinjured ventricles at 7 dpi, and ventricles treated with TC-E5002 uncovered three discrete transcriptional states, indicating a profound shift in gene-expression programs after TC-E5002 treatment (Figure 5A). Heat-map analysis revealed that TC-E5002 treatment partially reversed the cryoinjury-induced up-regulation of genes, likely due to the accumulation of the repressive histone mark H3K9me2 following *kdm7aa* blockade (Figure 5B). Examination of *kdm7aa*-dependent genes, whose transcripts were induced by injury but suppressed by TC-E5002 treatment, revealed strong enrichment for immune pathways (Figure 5C,D). GO terms highlighted cytokine activity, CXCR-family chemokine receptor binding, immune response and inflammatory response, while KEGG analysis underscored cytokine–cytokine receptor interaction together with Toll-like and NOD-like receptor signaling pathway. The Sankey diagram and associated heat map delineated individual genes driving these processes and visualized their expression profiles (Figure 5E,F). The consistent down-regulation of these modules following epigenetic blockade demonstrated that *kdm7aa* served as a direct activator of inflammatory programs essential for chemokine signaling and innate-immune circuits during ventricular repair. Subsequent UMAP analysis of the ligands selected from Figure 5F demonstrated that *cxcl8a*, *cxcl19*, *il1b*, and *tnfb* were specifically and highly enriched in cell population C3 (Figure 6A). Given the critical role of chemokines in neutrophil recruitment for cardiac regeneration, we performed qRT-PCR to examine the expression of *cxcl8a* and *cxcl19*. Consistent with transcriptomic data, both genes exhibited significant up-regulation in cryoinjured ventricles at 7 dpi, which was markedly suppressed by TC-E5002 treatment (Figure 6B,C). These results indicated that *kdm7aa* epigenetically regulated key chemokines to orchestrate inflammatory responses necessary for myocardial repair.

### 2.4. Kdm7a Upregulation Is Associated with Regenerative Capacity and Conserved in Mammalian Hearts

**Figure 7:**
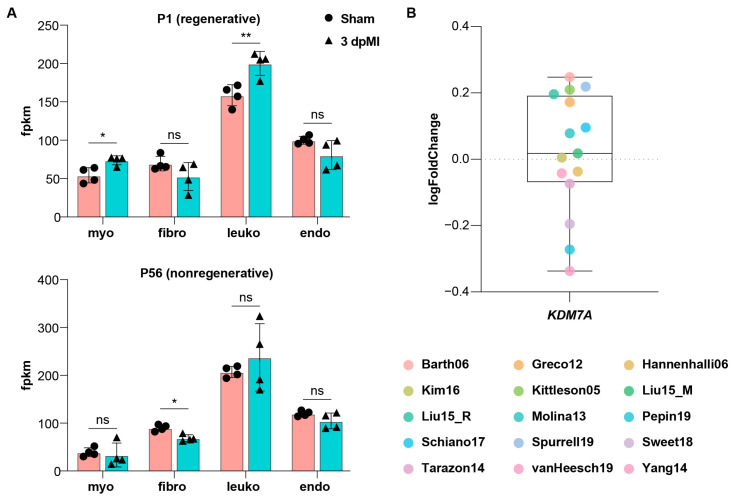
*Kdm7a* is upregulated after ventricular injury in neonatal but not adult mammals. (**A**) Reanalysis of cardiac regeneration RNA-seq datasets in P1 and P56 mice. Sham, sham-operated; dpMI, days post–myocardial infarction; myo, cardiomyocyte; fibro, fibroblast; leuko, leukocyte; endo, endothelial cell. Two-tailed Student’s *t*-test, ns, not significant, * *p* < 0.05, ** *p* < 0.01. (**B**) Reanalysis of 15 independent RNA-seq datasets from the ReHeaT database comparing failing and non-failing hearts. Each data point represents the fold change in KDM7A expression in failing versus non-failing hearts reported by the corresponding study.

Adult mammalian hearts have little regenerative capacity after injury, whereas neonatal hearts retain a brief regenerative window which is lost within the first postnatal week [12]. We reanalyzed the transcriptomic data by Quaife-Ryan et al., which profiled cardiomyocytes, fibroblasts, leukocytes, and endothelial cells isolated from sham-operated hearts and from hearts collected 3 days after myocardial infarction in neonatal (P1) and adult (P56) mice [13]. Interestingly, *Kdm7a* was significantly upregulated after injury in P1 cardiomyocytes, whereas no induction was detected in P56 mice (Figure 7A). We then examined *KDM7A* expression across 15 independent studies of failing and healthy human hearts in the ReHeaT database (https://saezlab.shinyapps.io/reheat/) and found no evidence of injury-associated induction in the non-regenerative human heart (Figure 7B). These observations support a conserved link between injury-induced *Kdm7a* upregulation and cardiac regenerative competence across mammals.

## 3. Discussion

MI is a leading cause of death and disability worldwide. In the adult human heart, ischemic injury causes irreversible cardiomyocyte loss and drives pathological remodeling and fibrosis [14]. Although the immune response is indispensable for initial clearance of necrotic tissue, its dysregulation exacerbates remodeling and scarring [15]. In contrast, zebrafish achieve lifelong, complete cardiac regeneration enabled by a well-organized immune program [16]. Defining the molecular cues that initiate and coordinate this pro-regenerative immunity is key to identify targets that enhance repair and limit fibrosis. Here, we linked epigenetic control to innate immune activation during zebrafish heart regeneration, identifying the histone demethylase Kdm7aa as a regeneration-induced factor selectively expressed in an immunomodulatory subset of ventricular cardiomyocytes. Functional and transcriptomic data analyses demonstrated that Kdm7aa orchestrated chemokine-mediated immune cell recruitment required for effective myocardial repair.

Single-cell RNA sequencing analysis revealed remarkable heterogeneity within the ventricular cardiomyocyte population during regeneration, identifying five distinct subpopulations with unique transcriptional signatures. The emergence of regeneration-specific clusters C3 and C5, characterized by inflammatory and remodeling gene programs, respectively, aligned with previous studies demonstrating the dynamic cardiomyocyte states following injury [9]. However, our study extended these observations, revealing that cardiomyocytes can adopt an immunomodulatory phenotype. They actively secreted chemokines and cytokines, acting as orchestrators of immune cell recruitment rather than merely responding to inflammatory signals. The specific expression of *kdm7aa* in the inflammatory cardiomyocyte cluster C3 suggested that epigenetic reprogramming enabled this phenotypic plasticity, allowing cardiomyocytes to acquire chemokine-secreting function during the regenerative process.

Previous studies on histone methylation in cardiac regeneration have largely focused on in vitro cardiomyocyte differentiation and reprogramming. Loss of the H3K27me2/3 demethylases *Kdm6a* or *Kdm6b* impaired embryonic stem cell differentiation toward the cardiac lineage [17,18]. In addition, the expression of *Kdm6a* and *Kdm6b* was up-regulated during the process of inducing fibroblasts to reprogram into cardiomyocytes, and their combined inhibition suppressed the cardiogenic transcription factors *Gata4*, *Mef2c*, and *Tbx5* in cardiomyocytes [19]. In contrast, the evidence that histone methylation regulated cardiac regeneration in vivo is still lacking. Induced expression of a mutant form of histone H3.3K27M during zebrafish heart regeneration decreased H3K27me3 in cardiomyocytes, enhanced sarcomere gene expression, and blocked cardiomyocyte dedifferentiation and proliferation [20]. Conversely, the H3K4 methyltransferase *smyd2* was upregulated after zebrafish cardiac injury and promoted cardiomyocyte proliferation via Stat3 phosphorylation [21]. Also, the Brg1-Kdm7aa-Notch signaling pathway within cardiac endothelial cells played a crucial role in governing myocardial regeneration through the modulation of H3K4me3 levels [22]. The identification of Kdm7aa as a regeneration-induced histone demethylase represented an advance in understanding the epigenetic landscape of cardiac repair. By re-examining the scRNA-seq data, we found that the *kdm7aa* was mainly upregulated in myocardial cells, and was also upregulated in endocardial cells, in a relatively minor proportion [9]. These results suggest that the *kdm7aa* may play important roles in both myocardial cells and endocardial cells. The temporal expression pattern of *kdm7aa*, peaking at 3–7 dpi, coincided with the critical window for immune cell infiltration and debris clearance, suggesting that *kdm7aa* functioned as a temporal regulator aligning inflammatory activation with regenerative demand [23]. Importantly, *Kdm7a* was up-regulated in neonatal but not adult mouse hearts after injury and failing human hearts. These observations supported the proposal that regenerative competence correlated with the capacity for transient, coordinated epigenetic remodeling, and further research is needed to validate its role in mammalian heart regeneration.

Our transcriptomic profiling revealed that *kdm7aa* governed a comprehensive inflammatory program centered on chemokine signaling, particularly through regulation of *cxcl8a* and *cxcl19*. These findings corroborated and extended previous work demonstrating that the Cxcl8a-Cxcr1 signaling pathway was the predominant pathway recruiting neutrophils to wounds and was essential for zebrafish heart regeneration [24,25]. However, further studies are needed to validate the role of *cxcl8a* and *cxcl19* in heart regeneration. Also, our study revealed that cardiomyocytes themselves, rather than epicardial cells or endocardial cells as previously assumed, served as sources of these chemokines during early regeneration [16,26]. The *kdm7aa*-dependent activation of multiple chemokine genes suggested a coordinated epigenetic program that ensured robust and sustained immune cell recruitment. Furthermore, the concurrent activation of Toll-like and NOD-like receptor signaling pathways indicated that *kdm7aa* enabled cardiomyocytes to sense damage-associated molecular patterns and mount an appropriate inflammatory response [27,28]. This dual capacity for damage sensing and chemokine production positioned *kdm7aa*-expressing cardiomyocytes as coordinators of the regenerative inflammatory milieu. The suppression of Kdm7aa by TC-E5002 treatment demonstrated the therapeutic potential of targeting KDM7 activity, though the timing and duration of such interventions would require careful optimization to avoid disrupting beneficial inflammation while preventing excessive scarring.

## 4. Materials and Methods

### 4.1. Zebrafish Husbandry

Wild-type zebrafish of Tübingen (TU) strain were maintained in a circulating aquaculture system at 28.5 °C under a 14 h/10 h light/dark photoperiod. Stocking density was kept below five adults per liter, and the fish were fed twice daily with live Artemia. All procedures were approved by the IACUC of Fujian Medical University (IACUC FJMU 2025-0077).

The transgenic line *Tg(vmhc:mCherry-NTR; amhc:EGFP)* was used to establish an embryonic zebrafish heart-injury model [29]. *kdm7aa* mosaic mutant embryos were generated by CRISPR/Cas9 system. Four single-guide RNAs (sgRNAs) were synthesized, transcribed in vitro as previously described, and co-injected with recombinant Cas9 protein into one-cell stage embryos [9]. The sgRNA target sites were: TCAGGTGACCAAACGCTTCT, GACATCTTTTGAGCGATATC, ACCTTGCACTATATGAGGCC, and TTGTCTGGCACTTCGCTGTT. For overexpression, *kdm7aa* mRNA was injected into fertilized eggs at a dosage of 50 ng per embryo.

### 4.2. Analysis of Single-Cell RNA-Seq Data

The scRNA-seq data were obtained from our previous reports [9]. Gene-expression matrices of the ventricular cardiomyocytes (N = 510) were log-normalized and filtered to retain cells with >1000 detected genes. Genes expressed in fewer than three cells (expression level ≤ 1) were excluded. Using Seurat v3.2.2, we identified the top 2000 variably expressed genes and performed principal-component analysis (PCA). Statistical significance of individual principal components (PCs) was assessed with JackStraw analysis, and the first 15 PCs were used for downstream Uniform Manifold Approximation and Projection (UMAP) analyses.

### 4.3. Chemical Treatment

*Tg(vmhc:mCherry-NTR; amhc:EGFP)* embryos at 3 days post-fertilization (dpf) were exposed to 5 mM metronidazole (MTZ; Merck, Darmstadt, Germany) for 4 h to induce cardiomyocyte ablation, as previously described [29]. At 4 days post-treatment (4 dpt), embryos were imaged, and the regeneration ratio was calculated for each treatment as the percentage of embryos that regained ventricular mCherry fluorescence. For bulk RNA-sequencing, adult zebrafish received daily intraperitoneal injections of 20 µL TC-E5002 (1.75 mM; Selleck, Houston, TX, USA) from 1 to 7 days post-injury (dpi).

### 4.4. Cryoinjury

Adult zebrafish were anesthetized by immersion in system water containing 0.02% (*w*/*v*) tricaine methanesulfonate (MS-222; Merck, Darmstadt, Germany). After complete anesthesia, zebrafish were positioned laterally on a moistened sponge. A small incision was made through the pericardial sac with fine micro-scissors. A pre-cooled cryoprobe was then applied to the exposed ventricular surface for 20 s, producing a focal freeze injury. Sham-operated controls underwent the identical surgical procedure, except omitting the cryoprobe process. Following surgery, the incision was gently closed and zebrafish were returned to fresh system water for recovery and daily monitoring. Hearts were excised at defined time points and rinsed in ice-cold phosphate-buffered saline (PBS) for downstream analyses.

### 4.5. Transcriptomic Analysis

Total RNA was isolated from sham-operated ventricles, 7 dpi ventricles, and TC-E5002-treated 7 dpi ventricles and submitted to SeqHealth Technology Co., Ltd. (Wuhan, China) for sequencing on an Illumina (San Diego, CA, USA) NovaSeq 6000 platform (paired-end, 150 bp). Each group contained 3 biological replicates, and each sample was sequenced to a depth of 6 Gb, as previously reported [30]. Differentially expressed genes (DEGs) were identified with the DESeq2 package (Version 1.38.3) [31]. The DEG was defined as genes with Foldchange > 2 and *p* value < 0.05. Gene Ontology (GO) and Kyoto Encyclopedia of Genes and Genomes (KEGG) pathway enrichment were analyzed with DAVID, and Sankey diagrams as well as gene-set enrichment analysis (GSEA) plots were generated using the Bioinformatics online suite (https://www.bioinformatics.com.cn) (accessed on 13 June 2025) [32].

To assess evolutionarily conserved regulation of *kdm7aa*, we re-analyzed single-cell RNA-seq data from neonatal and adult mouse hearts after injury (NCBI GEO accession GSE95755) [13]. In addition, *KDM7A* expression in human heart failure was evaluated using the ReHeaT compendium, which aggregates 15 independent bulk-transcriptome datasets comparing failing and non-failing human hearts (https://saezlab.shinyapps.io/reheat/) (accessed on 24 February 2023).

### 4.6. In Situ Hybridization

Control and MTZ-treated zebrafish embryos (4–7 dpf) were fixed overnight at 4 °C in 4% paraformaldehyde (PFA). After permeabilization with proteinase K and re-fixation in PFA, digoxigenin (DIG)-labeled antisense RNA probes were synthesized by in vitro transcription from linearized plasmid templates and hybridized to the embryos overnight at 65 °C in hybridization buffer. Following stringent post-hybridization washes, bound probes were detected with an alkaline phosphatase-conjugated anti-DIG antibody and visualized using an NBT/BCIP chromogenic substrate. Embryos were then post-fixed, cleared in glycerol, and imaged under identical microscopy settings.

### 4.7. Quantitative Real-Time PCR (qRT-PCR)

Total RNA was extracted from ventricular tissue harvested at 1, 3, 7, and 14 dpi using TRIzol reagent (Invitrogen, Carlsbad, CA, USA). One microgram of RNA was reverse-transcribed with the Evo M-MLV kit (Accurate Biology, Changsha, China) to generate first-strand cDNA. qRT-PCR was performed in three biological triplicates with SYBR Green Pro Taq HS premix (Accurate Biology, Changsha, China) on an Agilent AriaMX real-time PCR system (Santa Clara, CA, USA). Relative transcript levels were calculated with the 2^−ΔΔCt^ method and normalized to *actb2*.

### 4.8. Statistical Analysis

Statistical analyses were conducted in GraphPad Prism v9.0. Group differences were assessed by Student’s two-tailed *t*-test. The results were shown as the mean with the standard deviation (SD). Differences in treatments compared to the control group were defined as significant at *p* < 0.05 (*), *p* < 0.01 (**), or *p* < 0.001 (***).

## 5. Conclusions

Our study identified Kdm7aa as an important epigenetic regulator that enabled cardiomyocytes to adopt a transient inflammatory phenotype crucial for heart regeneration. The conservation of regeneration-associated *Kdm7a* upregulation in neonatal mice indicated that this pathway, while dormant in adult mammals, might be reactivatable through targeted epigenetic interventions. The discovery of this cardiomyocyte-intrinsic mechanism for orchestrating immune responses revealed unexpected plasticity in cardiomyocytes and suggested novel therapeutic avenues for enhancing mammalian heart repair.

## Data Availability

The RNA-seq data are available on the NCBI Sequence Read Archive (SRA) database: PRJNA128702. Other materials used in this study are available from the corresponding authors on reasonable request.

## Abbreviations

The following abbreviations are used in this manuscript:

MI	Myocardial infarction
KMT	Lysine methyltransferase
KDM	Lysine demethylase
sgRNA	Single-guide RNA
PC	Principal component
PCA	Principal-component analysis
UMAP	Uniform manifold approximation and projection
MTZ	Metronidazole
dpf	Day post-fertilization
dpt	Day post-treatment
dpi	Day post-injury
PBS	Phosphate-buffered saline
DEG	Differentially expressed gene
GO	Gene ontology
KEGG	Kyoto encyclopedia of genes and genomes
GSEA	Gene-set enrichment analysis
PFA	Paraformaldehyde
DIG	Digoxigenin
qRT-PCR	Quantitative real-time PCR
CM-V	Ventricular cardiomyocyte
CM-A	Atrial cardiomyocyte
EC	Endothelial cell
EP	Epicardial cell
EPDC	Epicardial-derived cell
scRNA-seq	Single-cell RNA sequencing
ECM	Extracellular matrix
